# Regulating the crosstalk between *Bifidobacterium* and the brain: a potential therapeutic strategy for Alzheimer’s disease

**DOI:** 10.3389/fimmu.2026.1706811

**Published:** 2026-02-03

**Authors:** Liting Peng, Zhiming Zhang, Yuan Hu, Huijia Chen, Yingru Tian, Hongyan Ling

**Affiliations:** 1 Department of Physiology, Hengyang Medical School, University of South China, Hengyang, Hunan, China; 2 Department of Anesthesiology, The First People’s Hospital of Chenzhou, The Chenzhou Affiliated Hospital, Hengyang Medical School, University of South China, Chenzhou, Hunan, China; 3 Department of Clinical College, Qujing University of Medicine & Health Sciences, Qujing, Yunnan, China

**Keywords:** Alzheimer’s disease, *Bifidobacterium*, brain-derived neurotrophic factor, neuroinflammation, neurotransmitter, short-chain fatty acid

## Abstract

Alzheimer’s disease (AD) is a common dementia in the elderly population, typically manifested through symptoms of cognitive impairment (CI) and memory loss. Pathologically, it is characterized by abnormally elevated levels of amyloid-β (Aβ) deposition and tau phosphorylation. Given the rapid rate of population aging, many scientists are investigating AD, focusing on its pathogenic mechanisms and potential treatments. Unfortunately, to date, no highly effective therapeutic strategies have emerged. Intriguingly, multiple studies have revealed alterations in the gut microbiome of individuals with AD, suggesting it may serve as a novel avenue for investigating AD pathogenesis. *Bifidobacterium*, a pivotal probiotic in the gastrointestinal tract, is crucial in upholding the equilibrium of gut flora. Notably, marked deficiencies in *Bifidobacterium* have been observed in the guts of AD patients, underscoring the potential of further inquiry into the impact of Bifidobacteria on AD via the gut-microbe-brain axis. However, current research on the mechanisms through which Bifidobacteria can alleviate AD is limited, warranting further investigation. This review examines Bifidobacterial alterations in Alzheimer’s disease patients and the underlying mechanisms, with the aim of evaluating their potential as a therapeutic strategy for Alzheimer’s disease.

## 1 Introduction

The therapeutic landscape for AD is largely defined by strategies targeting its core neuropathological features, such as cholinesterase inhibitors for symptom management, monoclonal antibodies against amyloid-beta (Aβ) plaques and p-tau (1–6). However, the clinical efficacy of these approaches in halting or reversing disease progression remains profoundly limited, primarily due to the multifactorial nature of AD pathogenesis and the failure of single-target therapies (7, 8). This critical gap highlights the urgent need to explore novel pathways that mediate neurodegeneration. A notable phenomenon in AD research has emerged from the growing recognition of the gut-brain axis, which positions the highly dynamic gut microbiota as a significant, modifiable influencer of brain homeostasis and neuroinflammation (9, 10).

The human gastrointestinal tract is an intricate and dense microbial ecosystem, consisting of approximately 10 to 100 trillion commensal microbial cells (9). Fascinatingly, the gut microbiota can secrete neurotransmitters, neuromodulators, and various metabolites derived from amino acids (11, 12). Within this context, mounting evidence consistently reports intestinal dysbiosis in AD patients, suggesting a strong association between altered microbial composition and neurodegenerative processes (13). The key point is that the reduction of *Bifidobacterium*, a crucial genus of probiotics in the gut, has become one of the significant microbial characteristics in AD (14, 15). This discovery is particularly important because the abundance of Bifidobacteria decreases sharply with age, which is a major non-genetic risk factor for AD (14, 16, 17). Furthermore, *Bifidobacterium* plays indispensable roles, including competitive exclusion of pathogens, fortifying the intestinal mucosal barrier, and acting as a primary producer of beneficial short-chain fatty acids (SCFAs) (18–20). The convergence of its age-related decline, its pronounced depletion in AD patients, and its crucial functions—particularly in regulating systemic inflammation and metabolic health—raises a critical and timely hypothesis: The loss of *Bifidobacterium* may not be merely a consequence of AD, but an active contributor to its pathogenesis, mediated through the gut-brain axis.

Therefore, the therapeutic potential of utilizing *Bifidobacterium* as a probiotic intervention to ameliorate AD pathology is gaining momentum (21). Current research indicates that its beneficial effects are mediated through several interconnected mechanisms: by restoring microbial stability to reduce the circulating pool of pro-inflammatory molecules (22–24); by reinforcing the gut epithelial integrity to prevent the translocation of bacterial endotoxins (e.g., Lipopolysaccharide, LPS), thereby mitigating peripheral and neuroinflammation (25–27); and by utilizing metabolites such as SCFAs to directly modulate microglia function, neurotrophic factor expression (e.g., BDNF), and neurotransmitter systems (24, 28–32). This review aims to critically synthesize the current mechanistic understanding of AD-related *Bifidobacterium* alterations and the evidence supporting *Bifidobacterium* supplementation as a novel, targeted strategy for AD intervention.

## 2 Bifidobacteria and AD

### 2.1 The decrease of *Bifidobacterium* in aging population

The gut microbiota is highly sensitive to our daily lifestyle, including diet, and sleep deprivation (33). The maintenance of gut flora homeostasis is intricately linked to overall physical well-being. Intriguingly, an expanding body of research has uncovered that the aging process significantly influences the composition of the gut microbial community (34, 35). Aging is a pivotal factor in the development of dementia, yet the complex interplay between microbes, aging, and dementia remains elusive. The connection between these three ideas remains to be fully elucidated. Understanding this relationship may enhance our comprehension of their collective influence on cognitive function and potentially inform strategies for mitigating age-related neurological decline.

**Figure 1:**
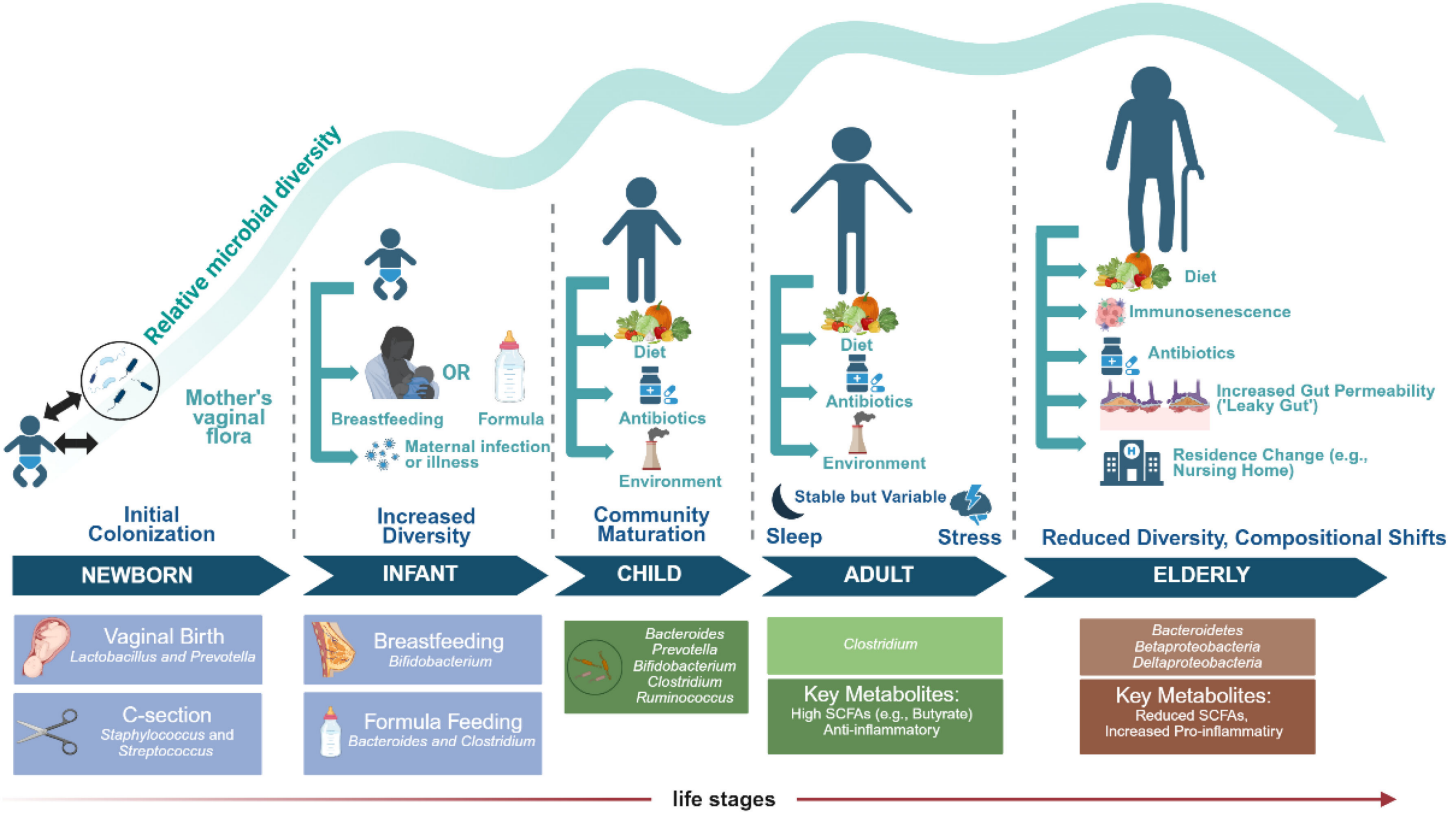
The composition of the human gut microbiota is dynamically modulated by various factors, including advancing age and alterations in lifestyle. (Image created using BioRender.com).

The gut microbiota exhibits substantial individual variation in infants, who acquire microbial communities akin to their mothers’ via multiple avenues, such as vaginal birth (36) and breastfeeding (37). The diversity of the human gut microbiota correspondingly increases with age. In a Japanese study, participants were categorized into three age cohorts-infants, adults, and the elderly-to investigate the alterations in intestinal microbiota across these distinct age groups (2). The study revealed a higher relative abundance of *Actinobacteria* in the infant cohort, an increased relative abundance of *Clostridia* in the adult group, and a significantly elevated prevalence of *Bacteroidetes, Betaproteobacteria*, and *Deltaproteobacteria* in the elderly group (**Figure 1**).

However, changes in gut microbiota cannot be ruled out by other factors, such as routine and diet. Therefore, whether age can be an independent factor influencing changes in gut flora is unclear for the time being and requires further research. Parker A et al. found that altering the gut flora can modulate biomarkers in the gut, eyes and brain (34). Transplantation of gut microbiota from young to aged mice modifies the microbial composition of the recipients, significantly increasing the relative abundance of taxa such as *Bifidobacterium animalis*, *Eubacterium* spp., *Akkermansia muciniphila*, and *Clostridium cocleatum*. At the same time, it reduces the expression of the inflammatory complement protein C3 in the retina and decreases the serum concentration of LPS-binding protein (LBP), thereby alleviating inflammation (34). Conversely, the transfer of fecal flora from older mice to young mice promotes microglia activation while exacerbating central nervous system (CNS) inflammation (34). In recent years, microglia overactivation has been denoted as an important factor in triggering neuroinflammation and an important cause of cognitive dysfunction (38). In contrast, altering the fecal flora of young mice reversed these deleterious effects. This may provide indirect evidence suggesting that the composition of the microbiota changes with age, thereby accelerating aging and cognitive decline. Therefore, the potential for using the significant decline of Bifidobacteria in the elderly gut as a novel therapeutic target for AD warrants further investigation (35).

### 2.2 Supplementation with *Bifidobacteria* can remodel gut microbiota in AD patients

Patients with AD exhibit clinical manifestations including CI and memory deficits, with the classic pathophysiological features characterized by Aβ deposition and hyperphosphorylation of tau protein. Nevertheless, an increasing body of evidence indicates disturbances in the gut microbiota of individuals with AD, including an elevated relative abundance of Mycobacterium avium, as well as a reduction in the relative abundance of *Bifidobacterium* and *Firmicutes* (29, 39, 40). To date, studies have not established that specific microbiota exert therapeutic effects in Alzheimer’s disease, underscoring a critical gap in the field. However, scientists are attempting to improve the imbalance of the microbiota by supplementing probiotics, ultimately alleviating AD.

This therapeutic potential of probiotics is not only limited to AD patients but also extends to the broader context of healthy aging. Shi S et al. demonstrated that oral administration of *Bifidobacterium longum* BB68S (BB68S, 5 × 10^10^ CFU/sachet)) daily for 8 weeks, markedly improved cognitive function in healthy older adults, as evidenced by an 18.89-point increase in the total RBANS score post-intervention (p<0.0001), with significant improvements observed in immediate memory, visuospatial/constructional skills, attention, and delayed memory (41). Concurrently, the BB68S treatment elevated the relative abundance of beneficial bacterial species, including *Lachnospira* and *Bifidobacterium*, while reducing the prevalence of taxa linked to cognitive impairment, such as *Collinsella* and *Parabacteroides* (41). The findings suggest that probiotic supplementation may mitigate cognitive deficits, but further verification is needed. The main limitations of this study include (42): a lack of analysis of the peripheral system and gut microbiota metabolites, making it unclear which metabolites were the source of therapeutic effects (43); although cognitive function significantly improved, changes in certain cognitive domains and the gut microbiota were not significant (44); an eight-week study may be insufficient to observe these outcomes, so longer-term research is needed (45); participants’ diets were not strictly controlled during the intervention, so we cannot be rule out the impact of dietary changes on the gut microbiome. Future studies need more rigorously designed research, such as strict dietary control.

Furthermore, the cognitive benefits of microbiota modulation can be enhanced through synbiotic approaches, which combine probiotics with prebiotics (46). A randomized, double-blind, placebo-controlled trial showed that a synbiotic supplement containing *Bifidobacterium animalis subsp. lactis* GCL2505 and inulin significantly improved overall cognitive function, including attention, cognitive flexibility, and executive function in elderly participants over 12 weeks (47). This intervention notably increased fecal *Bifidobacterium* counts and positively modulated several inflammatory markers, suggesting that the cognitive improvements were mediated by enhancing the gut environment and alleviating inflammation (More clinically relevant experiments are presented in **Table 1**).

**Table 1:** Partial Findings of Bifidobacteria Improvement in AD (Trial studies).

NO.	Disease	Intervention	CFU; Dose; Duration	linical Effects and Biological Observations of Intervention Group Compared with Placebo Group	Intestinal microorganisms	Trial ID; Reference
1	Older patients with mild cognitive impairment (MCI)	*B. animalis subsp. lactis* GCL2505	1 × 10^10^ CFU/day each; 12 weeks	The treatment group showed improvements in Cognitrax (short), MMSE-J, and MoCa-J scores.	Increased levels of fecal Bifidobacteria.	UMIN000048386; (47)
2	Healthy older adults	*B. longum* BB68S	5 × 10^10^ CFU/day each; 8 weeks	Significantly improves immediate memory and attention in healthy elderly individuals.	The relative abundance of the phyla Actinobacteria and Firmicutes increased in the BB68S group. The relative abundance of the phylum Proteobacteria decreased.	(41)
3	AD patients	*L. acidophilus*, *L. casei*, *B. bifidum*, and *L. fermentum*	2 × 10^9^ CFU/g for each; 12 weeks	Patients in the probiotic group showed improved MMSE scores (P < 0.001).		IRCT201511305623N60; (48)
4	AD patients	Selenium+*L. acidophilus*, *B. bifidum*, and *B. longum*	2 × 10^9^ CFU/day each; 12 weeks	The MMSE scores showed a significant improvement (P < 0.001). IL-8, TNF-α, and TGF-β levels decreased significantly.		IRCT20170612034497N5; (49)
5	AD patients	*B. longum subsp. Infantis* BLI-02, *B. breve* Bv-889, *B. animalis subsp. Lactis* CP-9, *B. bifidum* VDD088, and *Lactobacillus plantarum* PL-02	1 × 10^10^ CFU/day each; 12 weeks	There were no significant differences in cognitive test scores (ADAS-Cog, MMSE, ADL, and CDR) between the active control group and the treatment group. The treatment group exhibited a significant decrease in IL-1β, cortisol, MDA, and PCC levels, along with an increase in SOD activity (p < 0.05).	The treatment group experienced an increase in the abundance of *Bifidobacterium* (p = 0.317), Lactobacillus (p = 0.354), Ruminococcus (p = 0.286), Clostridium (p = 0.321), and Akkermansia (p = 0.934) at the genera level. Conversely. The presence of Megamonas (p = 0.213) decreased in the treatment group.	(50)
6	AD patients	*B. breve* A1	1×10^10^ CFU/day each; 24 weeks	Patients in the treatment group showed improvement in their MMSE scores.		NCT05145881; (51)
**7**	Older patients with MCI	*B. breve* MCC1274	2 × 10^10^ CFU/day each; 16 weeks	Patients in the treatment group showed improvement on the Assessment of Neuropsychological Status (RBANS) and the Japanese version of the MCI Screen (JMCIS) tests.		UMIN000037725; (52)
8	AD patients	*B. breve* MCC1274	2×10^10^ CFU/day each; 24 weeks	No significant difference was found between ADAS-Jcog and MMSE.		UMIN000031507; (53)

These studies provide evidence that *Bifidobacterium* supplements enhance cognition (41, 46). However, a critical question remains: How do these gut microbes exert profound effects on the brain? The mechanisms by which *Bifidobacterium* coordinates regulation of the gut-brain axis are only beginning to be elucidated. Unraveling these pathways is essential for translating probiotic therapies into targeted clinical interventions for AD.

## 3 Mechanisms of *Bifidobacteria* improving AD via the gut-brain axis

Increasing evidence suggests a communication between the nervous system and the gut, with the microbe-gut-brain axis emerging as a pivotal concept in the investigation of neurodegenerative diseases. This communication is mediated primarily through immune-related, neural, endocrine, and metabolic signaling pathways (54). Thus, the maintenance of gut microbiota homeostasis is essential for ensuring optimal brain function. *Bifidobacterium*, a commonly utilized probiotic, is frequently administered alone or in concert with other probiotics to modulate the intestinal flora environment for therapeutic purposes and is currently widely employed in the management of intestinal diseases, diabetes mellitus (55), and liver diseases (56). In light of the observation that AD patients exhibit reduced levels of Bifidobacteria within their intestinal microbiota, researchers have hypothesized that Bifidobacterial supplementation, alone or in combination with other strategies, may be a potential therapeutic approach for AD.

### 3.1 *Bifidobacteria* improved AD by neuroinflammation inhibition

**Figure 2:**
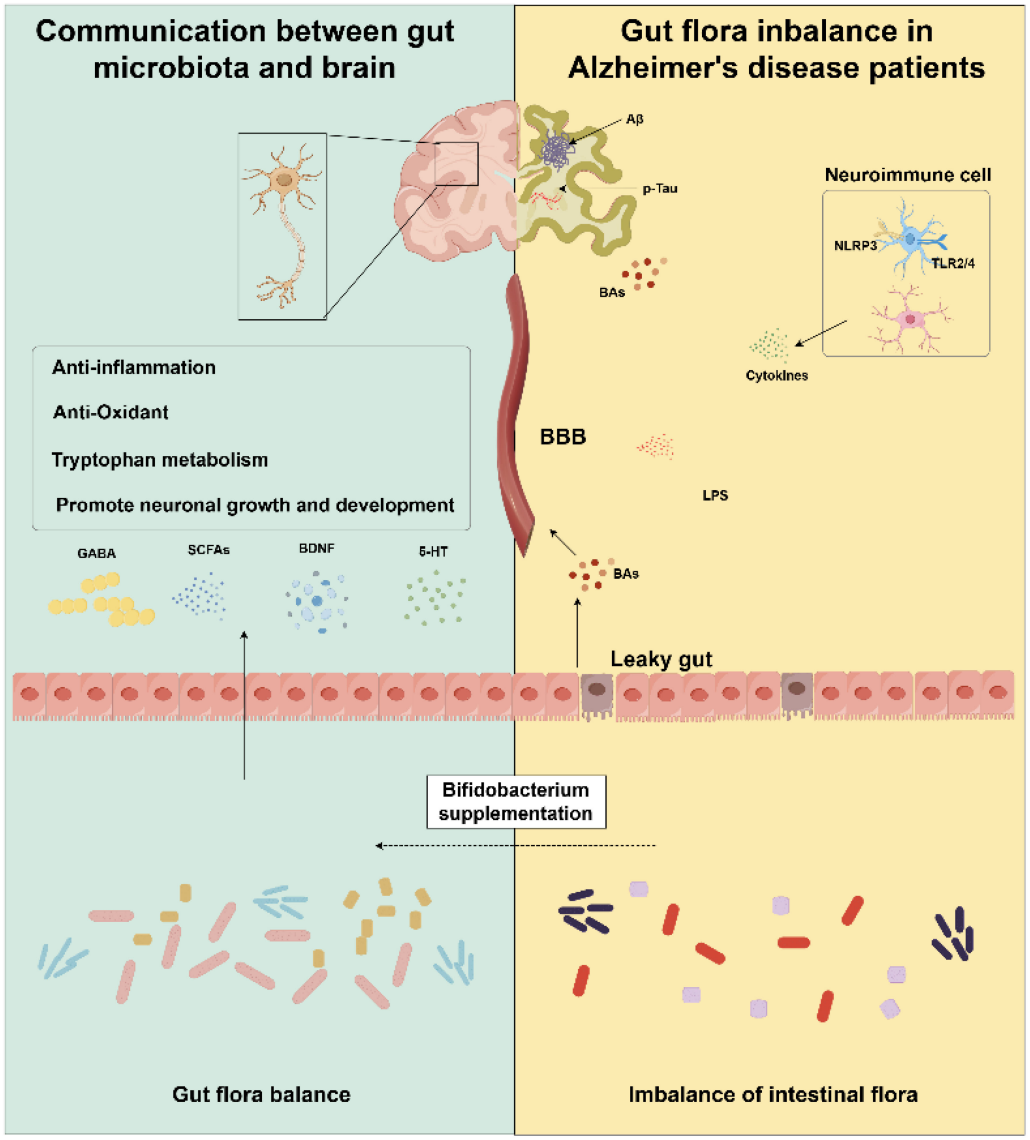
By supplementing Bifidobacteria, it can promote the production of GABA, SCFAs, BDNF and 5-HT, reduce neuroinflammation, promote the growth and development of neurons and thus improve AD. (Image created using Figdraw). GABA, gamma-aminobutyric acid; SCFAs, short-chain fatty acids; BDNF, Brain-derived neurotrophic factor; Aβ, amyloid-β; BAs, bile acids; LPS, Lipopolysaccharide; NLRP3, NOD-like receptor 3; TLR2/4, Toll like receptors 2/4.

Neuroinflammation is a key pathological feature of AD, and its origin is frequently traced to the gut (57). Disruption of the symbiotic relationship between the gut microbiota and the intestinal immune system can exacerbate inflammatory processes, characterized by the release of pro-inflammatory cytokines and increased intestinal permeability, often referred to as “leaky gut” (**Figure 2**) (58). Critically, this inflammation extends along the gut-brain axis, leading to neuroinflammation within the brain (39). On one hand, gut dysbiosis abnormally activates microglia, causing them to release chemokines and trigger neuroinflammation (59, 60); on the other hand, it also promotes the production of LPS, leading to intestinal inflammation (61). In populations with impaired gut and blood-brain barrier (BBB), such as the elderly, LPS more easily transfers to the brain (62). Notably, plasma LPS levels in Alzheimer’s disease patients are significantly higher than in healthy controls, contributing to a mild chronic inflammatory state in AD patients (63). Upon reaching the brain, LPS interacts with microglia, eliciting the activation of toll-like receptors (TLRs) and initiating potent neuroinflammatory responses (39).

Multiple studies have shown that administration of *Bifidobacterium* (as single strains or in composite formulations) significantly reduces the expression of pro-inflammatory factors like TNF-α and IL-1β in colonic tissue and decreases the infiltration of NF-κB+CD11c+ pro-inflammatory immune cells (64–66). This indicates that Bifidobacteria effectively alleviate local intestinal inflammation. Furthermore, by repairing the intestinal barrier and optimizing the microbiota composition, *Bifidobacterium* treatment leads to a significant decrease in LPS levels in both blood and feces (64, 66), signifying effective control of gut-derived systemic inflammation, or metabolic endotoxemia. This reduction in peripheral inflammatory signals subsequently extends to the CNS. Across multiple relevant models, *Bifidobacterium* intervention significantly decreased the expression of core pro-inflammatory factors—including TNF-α, IL-1β, and IL-6—in the hippocampus, a brain region critical for cognitive function, while concurrently inhibiting the activation of the NF-κB signaling pathway (64–66). This direct alleviation of the neuroinflammatory microenvironment represents a key mechanism by which Bifidobacteria may mitigate the progression of AD.

### 3.2 Bifidobacteria improves AD by increasing SCFAs

SCFAs are the primary metabolic products produced by the fermentation of dietary fiber by the gut microbiota. Among them, acetate, propionate, and butyrate are the most extensively studied and functionally important members. They are regarded as key “microbiota-host” communication molecules, playing a central role in neurotrophic regulation (67). As important acid-producing bacteria, *Bifidobacterium* positively influences gut-brain axis function by promoting the generation of SCFAs (68). These SCFAs can not only cross the BBB to directly act on the CNS but also indirectly regulate brain function and behavior through peripheral pathways (67). For instance, Fernando et al. found that supplementation with a composite formulation of *Bifidobacterium breve*, *Bifidobacterium longum*, and *Lactobacillus rhamnosus* significantly increased the total levels of SCFAs in the gut, highlighting the important role of *Bifidobacterium* in driving SCFA production (69).

*Bifidobacterium* primarily produces acetate and lactic acid through glucose metabolism, with acetate being its main end product (17). The mechanisms by which SCFAs ameliorate AD involve multiple pathways. Firstly, in maintaining the gut barrier function, acetate, as a vital energy source for colonic epithelial cells, supports their normal proliferation and function, thereby ensuring the integrity of the intestinal mucosa (70). More importantly, acetate can upregulate the expression and stability of tight junction (TJ) proteins (such as Claudin, Occludin, and ZO-1), reducing intestinal permeability (i.e., “leaky gut”). This effectively prevents harmful substances like endotoxins (e.g., LPS) from entering the bloodstream, thereby alleviating LPS-driven systemic and CNS inflammation and achieving indirect neuroprotection (71).

**Figure 3:**
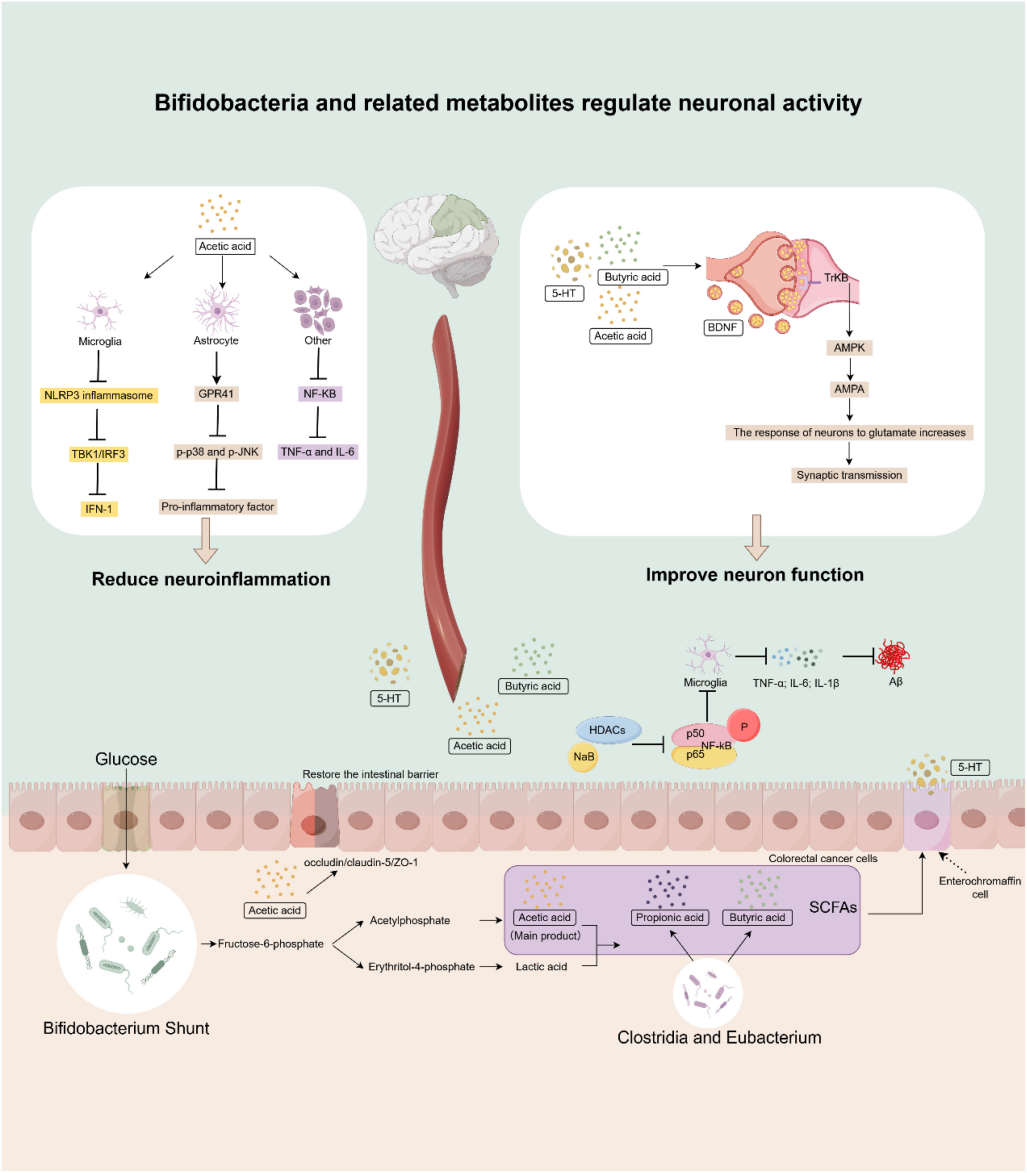
The mechanism by which Bifidobacteria and their related metabolites improve AD. The main product of SCFAs produced by Bifidobacteria is acetic acid. Acetic acid and lactic acid can serve as metabolic substrates for other microbial communities (Clostridia and Eubacterium), leading to the generation of propionic acid and butyric acid. Butyric acid, in turn, promotes the production of 5-HT in enterochromaffin cell. These metabolites improve AD by reducing the release of inflammatory factors, decreasing the production of Aβ, and enhancing neuronal function. (Image created using Figdraw).

Secondly, SCFAs, particularly butyrate, demonstrate potent direct neuroprotective effects (72). Although *Bifidobacterium* does not produce butyrate directly, its metabolic products, acetate and lactic acid, can serve as substrates for other butyrate-producing bacteria (such as *Clostridia* and *Eubacterium*), indirectly promoting butyrate generation through a “cross-feeding” mechanism (**Figure 3**) (73).

This synergistic acid production process is crucial for host health. In AD mouse models, intervention with sodium butyrate (NaB) not only significantly enhanced synaptic plasticity but also directly reduced the levels of pro-inflammatory cytokines (TNF-α, IL-6, IL-1β) in the brain, inhibited the overactivation of microglia, and prevented the deposition of β-amyloid (Aβ) (74). Its molecular mechanism involves binding to the GPR109A receptor, which in turn upregulates PPAR-γ expression and inhibits the activation of the TLR4/NF-κB signaling pathway. This process corrects the M1/M2 polarization imbalance in microglia, ultimately potentially improving cognitive deficits in mice (75). In addition to butyrate, acetate can also exert anti-neuroinflammatory effects by activating receptors such as GPR41 on microglia, thereby inhibiting pro-inflammatory signaling pathways like p38 MAPK, JNK, and NF-κB (76–78). The above studies exemplify the great potential of SCFAs and related salts, especially butyric acid and butyrate molecules, in improving AD.

In summary, current evidence strongly suggests that *Bifidobacterium* can exert its multifaceted functions by modulating the generation and composition of SCFAs. These functions include strengthening the intestinal barrier, exerting systemic anti-inflammatory effects, and providing direct neuroprotection, such as inhibiting neuroinflammation, reducing Aβ accumulation, and improving synaptic function. Therefore, targeting the *Bifidobacterium*-SCFA axis represents a highly promising therapeutic strategy for intervening in AD, and its associated molecular mechanisms provide an important theoretical foundation (79–85).

### 3.3 Bifidobacteria improved AD by increasing BDNF

Brain-derived neurotrophic factor (BDNF) is a critical protein essential for neuronal survival, growth, and function (86). Its promotion of neurogenesis and synaptic plasticity constitutes the biological basis of learning and memory. Substantial evidence indicates that BDNF levels in the brains of patients with AD are negatively correlated with disease severity (87, 88). In recent years, the modulation of BDNF-mediated brain function via the gut-brain axis has emerged as a significant candidate mechanism for probiotic intervention in neurodegenerative disorders (89). Although *Bifidobacterium* does not produce BDNF itself, multiple studies have demonstrated its capacity to significantly elevate BDNF levels in the host brain (90).

The mechanisms by which *Bifidobacterium* upregulates BDNF are multifaceted. At the molecular signaling level, BDNF activates its receptor, TrkB, to modulate core brain functions such as long-term potentiation (LTP), dendritic spine growth and maturation, and adult hippocampal neurogenesis (91, 92). At the epigenetic level, *Bifidobacterium* can promote BDNF expression by influencing histone modifications. For instance, *Bifidobacterium bifidum* BGN4 and *Bifidobacterium longum* BORI were shown to effectively enhance BDNF expression by increasing the trimethylation of histone H3 lysine 9 (H3K9me3), thereby ameliorating age-related cognitive deficits in mice (93).

However, a deeper upstream regulation underlying these mechanisms appears to be closely associated with the suppression of neuroinflammation driven by gut dysbiosis. Gut dysbiosis, particularly in aging or AD models, leads to an increased abundance of pro-inflammatory bacteria such as *Proteobacteria* and a concomitant rise in their LPS production (64). LPS can compromise the BBB through multiple mechanisms, thereby promoting the development of various cerebral diseases, such as sepsis-associated encephalopathy (SAE) (44). The primary way LPS disrupts the BBB is by inducing inflammation and directly acting on endothelial cells: LPS stimulates NF-κB expression by binding to Toll-like receptor 4 (TLR4) (94). The activated NF-κB pathway subsequently enhances BBB permeability by reducing the expression of TJ proteins, such as Occludin (95, 96). This damage to the barrier allows circulating LPS and harmful chemical substances to penetrate the CNS more easily (44), consequently inducing CNS dysfunction. Activated NF-κB not only induces the release of pro-inflammatory cytokines like TNF-α and IL-1β but also suppresses BDNF expression while upregulating key enzymes in the Aβ production pathway: β- and γ-secretases (97, 98). This creates a vicious cycle of “inflammation-amyloidosis-BDNF suppression,” ultimately leading to CI (99).

Against this background, numerous studies have provided direct evidence for the therapeutic effects of *Bifidobacterium*. For example, a combined intervention with *Bifidobacterium adolescentis* NK98 and *Lactobacillus reuteri* NK33 not only upregulated hippocampal BDNF expression but also synergistically inhibited the NF-κB signaling pathway, thereby mitigating neuroinflammation (100). Similarly, treatment with strains like *Bifidobacterium breve* CCFM1025 has been shown to enhance synaptic plasticity and elevate levels of BDNF and key synaptic proteins, including postsynaptic density protein 95 (PSD-95) (20). More recent studies have further elucidated the detailed mechanisms of this process. For instance, *Bifidobacterium longum* NK46, both alone and in combination with *Lactobacillus mucosae* NK41 (NKc), effectively suppressed the abundance of LPS-producing gut bacteria and reduced blood LPS levels, thereby blocking the aforementioned inflammatory cascade at its source (64). *In vivo* studies confirmed that oral administration of NK46 or NKc significantly inhibited NF-κB activation in the hippocampus of AD and aged mouse models (64, 101). This, in turn, relieved the suppression on BDNF, leading to a marked increase in hippocampal BDNF expression and the population of BDNF+ neurons, accompanied by an improvement in cognitive deficits (64, 99).

Notably, the benefits of *Bifidobacterium* also extend to associations with other pathological processes. For instance, in a model of CI induced by the periodontopathogen *Porphyromonas gingivalis*, a combination of *Bifidobacterium bifidum* NK391 and *Lactobacillus pentosus* NK357 similarly reversed the suppressed levels of hippocampal BDNF and N-methyl-D-aspartate receptor (NMDAR) by inhibiting the NF-κB pathway (66).

In conclusion, *Bifidobacterium* upregulates BDNF through a multi-layered mechanistic network. It may not only directly promote BDNF gene expression through epigenetic modifications (93), but more importantly, it alleviates the suppression of BDNF expression by reshaping the gut microbiota, inhibiting LPS production and absorption, and blocking NF-κB-mediated neuroinflammation. This process is tightly coupled with other functions, such as attenuating Aβ pathology and preserving synaptic function (64, 66, 91–93, 99, 102), providing a robust theoretical basis for improving AD.

### 3.4 Bifidobacteria improved AD by regulating neurotransmitters release

Neurotransmitters serve as fundamental mediators of interneuronal communication and are indispensable for the preservation of normal brain functionality (103, 104). These molecules not only facilitate the transmission of neural signals but also play a pivotal role in modulating cognitive processes, emotional responses, and behavioral outcomes (105). The objective of this section is to delineate the mechanisms by which Bifidobacteria modulate key neurotransmitters, including serotonin (5-HT), and gamma-aminobutyric acid (GABA), thereby exerting potential therapeutic effects on AD (**Figure 2**).

#### 3.4.1 5-HT

5-HT, a neurotransmitter extensively researched in the CNS, is involved in both central and peripheral physiological processes, modulating a diverse array of functions (105). For instance, in AD mouse models, the accumulation of Aβ correlates with a decrease in 5-HT2A receptor expression (106), whereas targeting 5-HT1B and 5-HT2C receptors has demonstrated the potential to mitigate Aβ neurotoxicity and tau hyperphosphorylation (107, 108). Notably, over 90% of the body’s 5-HT is synthesized by intestinal enterochromaffin (EC) cells, a process in which the gut microbiota and their metabolites, particularly SCFAs, are critical regulators (109). Studies have shown that these SCFAs, especially butyric acid, can directly stimulate EC cells, establishing a direct signaling axis within the “microbiota-gut-brain” communication network (110).

Based on this, *Bifidobacterium*, as a primary producer of SCFAs, is posited to have the potential to indirectly modulate central 5-HT levels (111–113). *Bifidobacterium animalis subsp. lactis* HN019 (*B. lactis* HN019TM) has been applied in infant foods and studies have demonstrated its benefits in maintaining normal physiological functions in the elderly, which may be related to the regulation of 5-HT signaling by SCFAs produced through microbial fermentation (19). Zhang S et al. supplemented APP/PS1 mice with prebiotics to stimulate the growth and development of gut probiotics, and found a significant increase in the relative abundance of Bifidobacteria, as well as alterations in the concentrations of neurotransmitters GABA and 5-HT in the brains of APP/PS1 mice, while reversing the cognitive dysfunction in APP/PS1 mice (114). Although this study employed prebiotics rather than direct probiotic supplementation, it provides crucial indirect evidence supporting a causal chain linking “increased *Bifidobacterium* abundance - elevated BDNF levels - amelioration of AD phenotypes.”

#### 3.4.2 GABA

GABA is the primary inhibitory neurotransmitter in the mammalian CNS (115). It mediates signaling between neurons, thereby modulating neuronal excitability by activating GABA receptors (116). In AD pathogenesis, GABAergic system dysregulation can lead to neuronal hyperexcitability, disrupted neural network activity, neuroinflammation, and synaptic impairment (117). Theoretically, restoring the excitatory-inhibitory balance by enhancing GABAergic signaling represents a viable therapeutic strategy for AD (118, 119). The gut microbiota is considered a significant peripheral source of GABA, with certain *Bifidobacterium* strains possessing the genetic basis to convert glutamate into GABA (120).

Emerging research indicates that GABA in the human gastrointestinal tract may originate from the conversion of dietary monosodium glutamate (MSG) by gut microbiota (31, 121). Nevertheless, the capacity of gut-derived *Bifidobacterium* strains to synthesize GABA has not been extensively investigated. Presently, the primary genes implicated in GABA synthesis in *Bifidobacterium* are gadB and gadC (30). The gadB gene codes for glutamate decarboxylase, an enzyme dependent on pyridoxal phosphate that catalyzes the decarboxylation of glutamate to yield GABA, while the gadC gene codes for the glutamate/GABA antiporter, a protein that facilitates the exchange of intracellular GABA for external glutamate. Consequently, Duranti S et al. conducted an analysis on *Bifidobacterium* strains expressing the gadB and gadC genes, utilizing data from the NCBI gene database, and identified 81 strains from 1002 Bifidobacteria encoding gadB and gadC, including *B. adolescentis, B. angulatum, B. dentium, B. merycicum, B. moukalabense, B. ruminantium*, and *B. samirii* (30). Notably, the *Bifidobacterium adolescentis* strain exhibited the highest expression of Gad genes within its genome, and *in vitro* studies revealed its capacity to effectively convert over 65% of its precursors to GABA, such as *B. adolescentis* PRL2019 and *B. adolescentis* HD17T2H, indicating that this taxon of *Bifidobacterium* is a potent GABA producer within the genus (30). In order to assess the efficacy of various probiotic strains in fermenting milk to enhance GABA production, Héctor Tamés et al. discovered, via computational simulations and *in vitro* experiments, that a particular strain, IPA60004, not only exhibited robust survival rates but also resulted in elevated GABA concentrations in their milk samples (122). E. Barrett et al. determined that, upon assessing 91 strains of bacteria originating from the human intestine, the GABA synthesized by Bifidobacteria displayed significant variability, with *Lactobacillus brevis* and *Bifidobacterium dentium* being the most efficient GABA producers (31). Studies investigating the application of Bifidobacteria for enhancing AD symptoms through GABA modulation are not without limitations. While *in vivo* experiments have demonstrated that intestinal GABA synthesis can be augmented by the addition of Bifidobacteria, which in turn may lead to improved cognitive performance in murine models (123–126), the exact mechanism underlying these effects has yet to be completely clarified. Additional research is required to delineate the sequential processes and the cellular and molecular dynamics at play.

Significant hurdles exist in the identification and selection of probiotics for AD therapy through the utilization of current databases and clinical samples. Some studies on the improvement of AD by *Bifidobacterium* are presented in **Table 2**. Existing databases and clinical sample collections frequently exhibit constraints in terms of size and geographic diversity, substantially impacting the generalizability and dependability of the research outcomes (30). These constraints highlight the necessity for broader and more varied datasets to inform the selection of probiotics that could be effective in AD therapy.

**Table 2:** Partial Findings of Bifidobacteria Improvement in AD (Animal studies).

No.	Bifidobacteria strains	Metabolites	Model	Findings/Mechanism	Intestinal microorganisms	Reference
1	*B. longum* NK46	BDNF	LPS-treated 5xFAD-transgenic mice Aged mice	Levels of Aβ, BACE, and Psen were all reduced. Y-maze task and other measures indicate improvements in cognitive and memory functions.	The population of Proteobacteria, Verrucomicrobia, Akkermansiaceae, Sutterellaceae, and Desulfovibrionaceae was decreased. The population of Odoribacteriaceae was increased.	(64)
2	Bifidobacteria		WT mice APP/PS1 mice	A significant decrease in the level of soluble Aβ in the cortex of AD + Bi mice.		(127)
3	*B. breve* MCC1274		WT mice	Reduce levels of senescin-1 protein, soluble Aβ42 in the hippocampus, and phosphorylated tau protein. Increase levels of synaptic proteins.		(87)
4	*B. bifidum* BGN4 and *B. longum* BORI	BDNF	Aged mice	Open field tests and other measures suggest improvements in cognitive function.	The abundance of Lachnoclostridium bacteria has decreased. The abundance of while that of Clostridium ASF356 has increased.	(93)
5	*B. bifidum* P61	BDNF	Aged mice	Y-maze task (YMT) suggests improvements in cognitive function.	The population of Odoribacteraceae and Deferribacteriaceae was decreased. The population of Akkermansiaceae and Bacteroidaceae was increased.	(65)
6	*B. bifidum* NK391	BDNF	CI mice	YMT and novel object recognition test (NORT) suggest improvements in cognitive function.	The population of Microbium genus, Erysipelothrix genus, and Acmannia genus was decreased.	(66)
7	*B*. CCFM1025	BDNF, acetate, and xanthurenic acid	5xFAD mice	Accumulation of Aβ1–42 was significantly reduced, and synaptic plasticity was enhanced. YMT suggests improvements in cognitive function;	The population of *Bifidobacterium*, Mucispirillum, and Ruminococcus was increased.	(21)
8	*B. breve* HNXY26M4	Acetate and butyrate	APP/PS1 mice	Accumulation of Aβ was significantly reduced. YMT and NORT suggest improvements in cognitive function.	The relative abundance of Lactobacillus and Acinetobacter was partially restored.	(128)
9	*B. bifidum* BGN4 and *B. longum* BORI	BDNF	AD mice	Reduced hippocampal neuronal death in 5xFAD mice. Restored BDNF and synaptic scaffolding proteins in the hippocampus of 5xFAD mice. Reduced Aβ42; YMT suggests improvements in cognitive function.	The population of Parvibacter, Incertae Sedis, and Oscillibacter was decreased. The population of *Bifidobacterium*, Akkermansia, Faecalibacterium, Erysipelothrix, and Candidatus Stoquefichus was increased.	(99)
10	*B. breve* CCFM1025	Acetate	Aβ1-42-treated mice	Reduce levels of Aβ1–42 in the hippocampus. YMT and other measures suggest improvements in cognitive function.	The relative abundance of Coprococcus was decreased. The relative abundance of Akkermansia was increased.	(129)
11	*B. bifidum* TMC3115		APP/PS1 mice		The relative abundance of Bacteroides was decreased. The relative abundance of Acetobacter and Bacteroides was increased.	(130)
12	*B. breve* MCC1274		AppNL-G-F mice	Reduces the production and deposition of β-amyloid in the hippocampus. Enhances ADAM10 protein levels in the hippocampal region of AppNL-G-F mice. Improves post-transcriptional regulation of ADAM10. Increases synaptic protein levels in the hippocampus. Does not alter the phosphorylation status of tau protein. NORT suggests improvements in cognitive function.	Not change.	(131)
13	*B. longum* NK46	BDNF	5XFAD-Tg and aged mice	Reduce the accumulation of Aβ plaques. YMT and other measures suggest improvements in cognitive function.	The relative abundance of thick-walled bacteria and amoebobacteria was decreased. The relative abundance of Bacteroides was increased.	(101)
14	*B. breve* A1	Acetate	Aβ25-35-treated mice	YMT and other measures suggest improvements in cognitive function.	The proportions of Faecalibacterium and Bacteroides were slightly lower. The proportions of Actinobacteria and Bifidobacteria significantly increased.	(132)
15	*B. breve* CCFM1025 and *B. breve* JSWX22M4	BDNF and acetate	Aβ1-42-treated mice	Reduce the accumulation of Aβ plaques. YMT and other measures suggest improvements in cognitive function.		(129)
16	*B. bifidum* BROB	BDNF	Aβ1-42-treated mice	passive avoidance test (shuttle box) suggests improvements in cognitive function.		(102)
17	*B. longum* NK173	BDNF	LPS-treated mice	YMT and other measures suggest improvements in cognitive function.	The relative abundance of phyla Firmicutes, Proteobacteria, and Proteobacteria was decreased. The relative abundance of phylum Bacteroidetes was increased.	(133)
18	*B. longum* NK219		LPS-treated mice	YMT and other measures suggest improvements in cognitive function.		(42)

## 5 Limitations

However, the “reconstruction” of the gut microbiota is not always complete or consistent. First, the extent of microbiota recovery is limited. In numerous studies, although favorable shifts in microbial composition occur, overall diversity and structure often do not fully restore to levels observed in healthy wild-type mice (130, 131, 133). The profound impact of AD pathology itself on the microbiota may make it difficult for simple supplementation with a few probiotics to fully reverse these changes (90). Second, strain- and host-specific effects are significant. Different *Bifidobacterium* strains (e.g., *B. longum* vs. *B. breve*) possess distinct metabolic characteristics and colonization abilities, leading to varying patterns of induced microbiota changes (133). Similarly, AD model mice with different genetic backgrounds exhibit distinct baseline microbiomes and responses to probiotics (64, 87, 102, 134), complicating direct comparison and extrapolation of research findings.

A key piece of evidence comes from the study by Hongwon Kim et al. (93). This study ingeniously compared the effects of probiotics (*B. bifidum* BGN4 and *B. longum* BORI) on young (3-month-old) and aged (16-month-old) mice. Results revealed that probiotic intervention significantly altered fecal microbiota composition in young mice but had minimal effect on elderly mice. However, in stark contrast to this “non-responsive” microbiota, probiotics similarly reversed DNA damage and apoptosis in elderly mice while improving age-related cognitive and memory deficits. This indicates that while *Bifidobacterium* supplementation consistently demonstrates positive effects on cognitive enhancement and neuroinflammation suppression, its capacity to remodel the gut microbiota is far from universal or uniform. Instead, it exhibits significant model dependency and age-related variability, constituting a prominent and central contradiction in current research (41, 47, 93).

Another core contradiction is the dissociation between cognitive improvement and core pathological changes. For instance, studies have observed enhanced performance in AD mice on water maze tests following supplementation with specific Bifidobacteria. However, some investigations do not demonstrate concurrent reductions in Aβ plaque burden or tau phosphorylation levels (50, 65, 135), particularly lacking corresponding data from human brains. Therefore, existing evidence remains insufficient to strongly demonstrate a direct link between *Bifidobacterium*-mediated cognitive improvement and core AD pathological products. A more plausible mechanism involves cognitive enhancement through pathways such as alleviating neuroinflammation, enhancing synaptic plasticity, or regulating neurotransmitter metabolism (41, 42, 47, 66, 128). This suggests that the “downstream effects” of microbiota restoration may be more complex than anticipated, with cognitive improvements resulting from multifactorial interactions rather than being an inevitable consequence of a single pathological marker’s improvement.

Furthermore, a significant challenge to the long-term efficacy of this approach is the transient nature of the microbial changes. Upon discontinuing probiotic supplementation, the gut microbiota of mice often reverts to its pre-intervention state within weeks (136–138). This indicates that oral administration of live bacteria alone may fail to achieve sustained microbial colonization and ecological reconstruction. The observed improvements are more likely a form of “functional regulation.” During their transient presence in the gut, probiotics exert beneficial physiological effects through their metabolites (e.g., short-chain fatty acids) or interactions with the host immune system, rather than fundamentally altering the long-term structural composition of the microbiota (139–142). This also explains why some studies require long-term, continuous administration to sustain effects.

## 6 Conclusion and future prospects

This review has synthesized the current evidence linking gut Bifidobacteria to Alzheimer’s disease (AD), revealing a landscape of promising therapeutic potential overshadowed by significant complexities and contradictions (143). While studies consistently report cognitive benefits associated with *Bifidobacterium* supplementation (51, 55, 129, 135), critical gaps remain, including the dissociation between cognitive improvement and core AD pathology, the strain- and host-specific nature of the effects, and the transient nature of microbial changes.

To transform these correlational insights into causative therapies, the field must evolve. Future research must prioritize establishing direct causality, moving beyond association. This will require innovative models, such as germ-free AD mice colonized with defined microbial communities, to dissect the precise role of *Bifidobacterium* and its key metabolites (e.g., specific SCFAs, tryptophan derivatives) in modulating neuroinflammation and synaptic function (17, 78, 144). Concurrently, critical, understudied parameters must be systematically addressed. Research must determine the optimal timing of intervention (pre-symptomatic vs. symptomatic stages), evaluate the synergistic potential of multi-strain formulations versus single strains, and elucidate the complex interactions with host diet and genetics. Finally, to bridge the translational gap, future clinical trials must be designed with enhanced rigor. This necessitates the incorporation of standardized cognitive endpoints, robust biomarker panels (e.g., neuroimaging, fluid biomarkers), and high-resolution metagenomic sequencing. By embracing these more sophisticated and mechanistic approaches, we can move from merely observing benefits to truly understanding and harnessing the microbiota-gut-brain axis for the development of effective, novel therapeutics against AD.

## References

[1] Scheltens P De Strooper B Kivipelto M Holstege H Chetelat G Teunissen CE . Alzheimer’s disease. Lancet. (2021) 397:1577–90. doi: 10.1016/S0140-6736(24)01883-X, PMID: 33667416 PMC8354300

[2] Odamaki T Kato K Sugahara H Hashikura N Takahashi S Xiao JZ . Age-related changes in gut microbiota composition from newborn to centenarian: a cross-sectional study. BMC Microbiol. (2016) 16:90. doi: 10.1186/s12866-016-0708-5, PMID: 27220822 PMC4879732

[3] Heneka MT Morgan D Jessen F . Passive anti-amyloid beta immunotherapy in Alzheimer’s disease-opportunities and challenges. Lancet. (2024) 404:2198–208. doi: 10.1016/S0140-6736(24)01883-X, PMID: 39549715

[4] Srivastava S Ahmad R Khare SK . Alzheimer’s disease and its treatment by different approaches: A review. Eur J Med Chem. (2021) 216:113320. doi: 10.1016/j.ejmech.2021.113320, PMID: 33652356

[5] Zhang D Zhang W Ming C Gao X Yuan H Lin X . P-tau217 correlates with neurodegeneration in Alzheimer’s disease, and targeting p-tau217 with immunotherapy ameliorates murine tauopathy. Neuron. (2024) 112:1676–93.e12. doi: 10.1016/j.neuron.2024.02.017, PMID: 38513667

[6] Zhang H Wang Y Wang Y Li X Wang S Wang Z . Recent advance on carbamate-based cholinesterase inhibitors as potential multifunctional agents against Alzheimer’s disease. Eur J Med Chem. (2022) 240:114606. doi: 10.1016/j.ejmech.2022.114606, PMID: 35858523

[7] Claesson MJ Jeffery IB Conde S Power SE O’Connor EM Cusack S . Gut microbiota composition correlates with diet and health in the elderly. Nature. (2012) 488:178–84. doi: 10.1038/nature11319, PMID: 22797518

[8] Ghosh TS Das M Jeffery IB O’Toole PW . Adjusting for age improves identification of gut microbiome alterations in multiple diseases. Elife. (2020) 9:e50240. doi: 10.7554/eLife.50240, PMID: 32159510 PMC7065848

[9] Ursell LK Metcalf JL Parfrey LW Knight R . Defining the human microbiome. Nutr Rev. (2012) 70:S38–44. doi: 10.1111/j.1753-4887.2012.00493.x, PMID: 22861806 PMC3426293

[10] Zang Y Lai X Li C Ding D Wang Y Zhu Y . The role of gut microbiota in various neurological and psychiatric disorders-an evidence mapping based on quantified evidence. Mediators Inflamm. (2023) 2023:5127157. doi: 10.1155/2023/5127157, PMID: 36816743 PMC9936509

[11] Angelucci F Cechova K Amlerova J Hort J . Antibiotics, gut microbiota, and Alzheimer’s disease. J Neuroinflamm. (2019) 16:108. doi: 10.1186/s12974-019-1494-4, PMID: 31118068 PMC6530014

[12] Dinan TG Cryan JF . The microbiome-gut-brain axis in health and disease. Gastroenterol Clin North Am. (2017) 46:77–89. doi: 10.1016/j.gtc.2016.09.007, PMID: 28164854

[13] Grabrucker S Marizzoni M Silajdzic E Lopizzo N Mombelli E Nicolas S . Microbiota from Alzheimer’s patients induce deficits in cognition and hippocampal neurogenesis. Brain. (2023) 146:4916–34. doi: 10.1093/brain/awad303, PMID: 37849234 PMC10689930

[14] Derrien M Turroni F Ventura M Van Sinderen D . Insights into endogenous Bifidobacterium species in the human gut microbiota during adulthood. Trends Microbiol. (2022) 30:940–7. doi: 10.1016/j.tim.2022.04.004, PMID: 35577716

[15] Xiao Y Zhao J Zhang H Zhai Q Chen W . Mining Lactobacillus and Bifidobacterium for organisms with long-term gut colonization potential. Clin Nutr. (2020) 39:1315–23. doi: 10.1016/j.clnu.2019.05.014, PMID: 31174942

[16] Shen H Zhao Z Zhao Z Chen Y Zhang L . Native and engineered probiotics: promising agents against related systemic and intestinal diseases. Int J Mol Sci. (2022) 23:594. doi: 10.3390/ijms23020594, PMID: 35054790 PMC8775704

[17] Markowiak-Kopec P Slizewska K . The effect of probiotics on the production of short-chain fatty acids by human intestinal microbiome. Nutrients. (2020) 12:1107. doi: 10.3390/nu12041107, PMID: 32316181 PMC7230973

[18] Gavzy SJ Kensiski A Lee ZL Mongodin EF Ma B Bromberg JS . Bifidobacterium mechanisms of immune modulation and tolerance. Gut Microbes. (2023) 15:2291164. doi: 10.1080/19490976.2023.2291164, PMID: 38055306 PMC10730214

[19] Cheng J Laitila A Ouwehand AC . Bifidobacterium animalis subsp. lactis HN019 Effects on Gut Health: A Review. Front Nutr. (2021) 8:790561. doi: 10.3389/fnut.2021.790561, PMID: 34970580 PMC8712437

[20] Abdulqadir R Engers J Al-Sadi R . Role of bifidobacterium in modulating the intestinal epithelial tight junction barrier: current knowledge and perspectives. Curr Dev Nutr. (2023) 7:102026. doi: 10.1016/j.cdnut.2023.102026, PMID: 38076401 PMC10700415

[21] Zhu G Guo M Zhao J Zhang H Wang G Chen W . Bifidobacterium breve intervention combined with environmental enrichment alleviates cognitive impairment by regulating the gut microbiota and microbial metabolites in Alzheimer’s disease mice. Front Immunol. (2022) 13:1013664. doi: 10.3389/fimmu.2022.1013664, PMID: 36203603 PMC9530393

[22] Zheng X Cai X Hao H . Emerging targetome and signalome landscape of gut microbial metabolites. Cell Metab. (2022) 34:35–58. doi: 10.1016/j.cmet.2021.12.011, PMID: 34986337

[23] Knox EG Aburto MR Clarke G Cryan JF O’Driscoll CM . The blood-brain barrier in aging and neurodegeneration. Mol Psychiatry. (2022) 27:2659–73. doi: 10.1038/s41380-022-01511-z, PMID: 35361905 PMC9156404

[24] Faulin T Estadella D . Alzheimer’s disease and its relationship with the microbiota-gut-brain axis. Arq Gastroenterol. (2023) 60:144–54. doi: 10.1590/s0004-2803.202301000-17, PMID: 37194773

[25] Bairamian D Sha S Rolhion N Sokol H Dorothee G Lemere CA . Microbiota in neuroinflammation and synaptic dysfunction: a focus on Alzheimer’s disease. Mol Neurodegener. (2022) 17:19. doi: 10.1186/s13024-022-00522-2, PMID: 35248147 PMC8898063

[26] Desbonnet L Garrett L Clarke G Bienenstock J Dinan TG . The probiotic Bifidobacteria infantis: An assessment of potential antidepressant properties in the rat. J Psychiatr Res. (2008) 43:164–74. doi: 10.1016/j.jpsychires.2008.03.009, PMID: 18456279

[27] Li J Wang J Wang M Zheng L Cen Q Wang F . Bifidobacterium: a probiotic for the prevention and treatment of depression. Front Microbiol. (2023) 14:1174800. doi: 10.3389/fmicb.2023.1174800, PMID: 37234527 PMC10205982

[28] Zhao Y Jaber V Lukiw WJ . Secretory products of the human GI tract microbiome and their potential impact on alzheimer’s disease (AD): detection of lipopolysaccharide (LPS) in AD hippocampus. Front Cell Infect Microbiol. (2017) 7:318. doi: 10.3389/fcimb.2017.00318, PMID: 28744452 PMC5504724

[29] Kesika P Suganthy N Sivamaruthi BS Chaiyasut C . Role of gut-brain axis, gut microbial composition, and probiotic intervention in Alzheimer’s disease. Life Sci. (2021) 264:118627. doi: 10.1016/j.lfs.2020.118627, PMID: 33169684

[30] Duranti S Ruiz L Lugli GA Tames H Milani C Mancabelli L . Bifidobacterium adolescentis as a key member of the human gut microbiota in the production of GABA. Sci Rep. (2020) 10:14112. doi: 10.1038/s41598-020-70986-z, PMID: 32839473 PMC7445748

[31] Barrett E Ross RP O’Toole PW Fitzgerald GF Stanton C . gamma-Aminobutyric acid production by culturable bacteria from the human intestine. J Appl Microbiol. (2012) 113:411–7. doi: 10.1111/j.1365-2672.2012.05344.x, PMID: 22612585

[32] Berger K Burleigh S Lindahl M Bhattacharya A Patil P Stalbrand H . Xylooligosaccharides increase bifidobacteria and lachnospiraceae in mice on a high-fat diet, with a concomitant increase in short-chain fatty acids, especially butyric acid. J Agric Food Chem. (2021) 69:3617–25. doi: 10.1021/acs.jafc.0c06279, PMID: 33724030 PMC8041301

[33] Askarova S Umbayev B Masoud AR Kaiyrlykyzy A Safarova Y Tsoy A . The links between the gut microbiome, aging, modern lifestyle and alzheimer’s disease. Front Cell Infect Microbiol. (2020) 10:104. doi: 10.3389/fcimb.2020.00104, PMID: 32257964 PMC7093326

[34] Parker A Romano S Ansorge R Aboelnour A Le Gall G Savva GM . Fecal microbiota transfer between young and aged mice reverses hallmarks of the aging gut, eye, and brain. Microbiome. (2022) 10:68. doi: 10.1186/s40168-022-01243-w, PMID: 35501923 PMC9063061

[35] Hopkins MJ Macfarlane GT . Changes in predominant bacterial populations in human faeces with age and with Clostridium difficile infection. J Med Microbiol. (2002) 51:448–54. doi: 10.1099/0022-1317-51-5-448, PMID: 11990498

[36] Dominguez-Bello MG Costello EK Contreras M Magris M Hidalgo G Fierer N . Delivery mode shapes the acquisition and structure of the initial microbiota across multiple body habitats in newborns. Proc Natl Acad Sci U S A. (2010) 107:11971–5. doi: 10.1073/pnas.1002601107, PMID: 20566857 PMC2900693

[37] Bezirtzoglou E Tsiotsias A Welling GW . Microbiota profile in feces of breast- and formula-fed newborns by using fluorescence in *situ* hybridization (FISH). Anaerobe. (2011) 17:478–82. doi: 10.1016/j.anaerobe.2011.03.009, PMID: 21497661

[38] Merighi S Nigro M Travagli A Gessi S . Microglia and alzheimer’s disease. Int J Mol Sci. (2022) 23:12990. doi: 10.3390/ijms232112990, PMID: 36361780 PMC9657945

[39] Megur A Baltriukiene D Bukelskiene V Burokas A . The microbiota-gut-brain axis and alzheimer’s disease: neuroinflammation is to blame? Nutrients. (2020) 13:37. doi: 10.3390/nu13010037, PMID: 33374235 PMC7824474

[40] Liu S Gao J Zhu M Liu K Zhang HL . Gut microbiota and dysbiosis in alzheimer’s disease: implications for pathogenesis and treatment. Mol Neurobiol. (2020) 57:5026–43. doi: 10.1007/s12035-020-02073-3, PMID: 32829453 PMC7541367

[41] Shi S Zhang Q Sang Y Ge S Wang Q Wang R . Probiotic bifidobacterium longum BB68S improves cognitive functions in healthy older adults: A randomized, double-blind, placebo-controlled trial. Nutrients. (2022) 15:51. doi: 10.3390/nu15010051, PMID: 36615708 PMC9824790

[42] Ma X Shin YJ Jang HM Joo MK Yoo JW Kim DH . Lactobacillus rhamnosus and Bifidobacterium longum alleviate colitis and cognitive impairment in mice by regulating IFN-gamma to IL-10 and TNF-alpha to IL-10 expression ratios. Sci Rep. (2021) 11:20659. doi: 10.1038/s41598-021-00096-x, PMID: 34667205 PMC8526673

[43] Graff-Radford J Yong KXX Apostolova LG Bouwman FH Carrillo M Dickerson BC . New insights into atypical Alzheimer’s disease in the era of biomarkers. Lancet Neurol. (2021) 20:222–34. doi: 10.1016/S1474-4422(20)30440-3, PMID: 33609479 PMC8056394

[44] Peng X Luo Z He S Zhang L Li Y . Blood-brain barrier disruption by lipopolysaccharide and sepsis-associated encephalopathy. Front Cell Infect Microbiol. (2021) 11:768108. doi: 10.3389/fcimb.2021.768108, PMID: 34804998 PMC8599158

[45] Zhang T Gao G Kwok LY Sun Z . Gut microbiome-targeted therapies for Alzheimer’s disease. Gut Microbes. (2023) 15:2271613. doi: 10.1080/19490976.2023.2271613, PMID: 37934614 PMC10631445

[46] Robinson GE Stewart DE . Postpartum psychiatric disorders. CMAJ. (1986) 134:31–7. PMC1490588 3510069

[47] Azuma N Mawatari T Saito Y Tsukamoto M Sampei M Iwama Y . Effect of continuous ingestion of bifidobacteria and dietary fiber on improvement in cognitive function: A randomized, double-blind, placebo-controlled trial. Nutrients. (2023) 15:4175. doi: 10.3390/nu15194175, PMID: 37836458 PMC10574581

[48] Akbari E Asemi Z Daneshvar Kakhaki R Bahmani F Kouchaki E Tamtaji OR . Effect of probiotic supplementation on cognitive function and metabolic status in alzheimer’s disease: A randomized, double-blind and controlled trial. Front Aging Neurosci. (2016) 8:256. doi: 10.3389/fnagi.2016.00256, PMID: 27891089 PMC5105117

[49] Tamtaji OR Heidari-Soureshjani R Mirhosseini N Kouchaki E Bahmani F Aghadavod E . Probiotic and selenium co-supplementation, and the effects on clinical, metabolic and genetic status in Alzheimer’s disease: A randomized, double-blind, controlled trial. Clin Nutr. (2019) 38:2569–75. doi: 10.1016/j.clnu.2018.11.034, PMID: 30642737

[50] Hsu YC Huang YY Tsai SY Kuo YW Lin JH Ho HH . Efficacy of probiotic supplements on brain-derived neurotrophic factor, inflammatory biomarkers, oxidative stress and cognitive function in patients with alzheimer’s dementia: A 12-week randomized, double-blind active-controlled study. Nutrients. (2023) 16:16. doi: 10.3390/nu16010016, PMID: 38201846 PMC10780998

[51] Kobayashi Y Kinoshita T Matsumoto A Yoshino K Saito I Xiao JZ . Bifidobacterium breve A1 supplementation improved cognitive decline in older adults with mild cognitive impairment: an open-label, single-arm study. J Prev Alzheimers Dis. (2019) 6:70–5. doi: 10.14283/jpad.2018.32, PMID: 30569089 PMC12280755

[52] Xiao J Katsumata N Bernier F Ohno K Yamauchi Y Odamaki T . Probiotic bifidobacterium breve in improving cognitive functions of older adults with suspected mild cognitive impairment: A randomized, double-blind, placebo-controlled trial. J Alzheimers Dis. (2020) 77:139–47. doi: 10.3233/JAD-200488, PMID: 32623402 PMC7592675

[53] Asaoka D Xiao J Takeda T Yanagisawa N Yamazaki T Matsubara Y . Effect of probiotic bifidobacterium breve in improving cognitive function and preventing brain atrophy in older patients with suspected mild cognitive impairment: results of a 24-week randomized, double-blind, placebo-controlled trial. J Alzheimers Dis. (2022) 88:75–95. doi: 10.3233/JAD-220148, PMID: 35570493 PMC9277669

[54] Sorboni SG Moghaddam HS Jafarzadeh-Esfehani R Soleimanpour S . A comprehensive review on the role of the gut microbiome in human neurological disorders. Clin Microbiol Rev. (2022) 35:e0033820. doi: 10.1128/CMR.00338-20, PMID: 34985325 PMC8729913

[55] Wang CH Yen HR Lu WL Ho HH Lin WY Kuo YW . Adjuvant Probiotics of Lactobacillus salivarius subsp. salicinius AP-32, L. johnsonii MH-68, and Bifidobacterium animalis subsp. lactis CP-9 Attenuate Glycemic Levels and Inflammatory Cytokines in Patients With Type 1 Diabetes Mellitus. Front Endocrinol (Laus). (2022) 13:754401. doi: 10.3389/fendo.2022.754401, PMID: 35299968 PMC8921459

[56] Lopez-Escalera S Lund ML Hermes GDA Choi BS Sakamoto K Wellejus A . *In vitro* screening for probiotic properties of lactobacillus and bifidobacterium strains in assays relevant for non-alcoholic fatty liver disease prevention. Nutrients. (2023) 15:2361. doi: 10.3390/nu15102361, PMID: 37242245 PMC10224198

[57] Thakur S Dhapola R Sarma P Medhi B Reddy DH . Neuroinflammation in alzheimer’s disease: current progress in molecular signaling and therapeutics. Inflammation. (2023) 46:1–17. doi: 10.1007/s10753-022-01721-1, PMID: 35986874

[58] Luo Y Tong Y Wu L Niu H Li Y Su LC . Alteration of gut microbiota in individuals at high-risk for rheumatoid arthritis associated with disturbed metabolome and the initiation of arthritis through the triggering of mucosal immunity imbalance. Arthritis Rheumatol. (2023) 75:1736–48. doi: 10.1002/art.42616, PMID: 37219936

[59] Woodburn SC Bollinger JL Wohleb ES . The semantics of microglia activation: neuroinflammation, homeostasis, and stress. J Neuroinflamm. (2021) 18:258. doi: 10.1186/s12974-021-02309-6, PMID: 34742308 PMC8571840

[60] Li H Xiang Y Zhu Z Wang W Jiang Z Zhao M . Rifaximin-mediated gut microbiota regulation modulates the function of microglia and protects against CUMS-induced depression-like behaviors in adolescent rat. J Neuroinflamm. (2021) 18:254. doi: 10.1186/s12974-021-02303-y, PMID: 34736493 PMC8567657

[61] Candelli M Franza L Pignataro G Ojetti V Covino M Piccioni A . Interaction between lipopolysaccharide and gut microbiota in inflammatory bowel diseases. Int J Mol Sci. (2021) 17:7689. doi: 10.3390/ijms22126242, PMID: 34200555 PMC8226948

[62] Sanchez-Tapia M Mimenza-Alvarado A Granados-Dominguez L Flores-Lopez A Lopez-Barradas A Ortiz V . The Gut Microbiota-Brain Axis during Aging, Mild Cognitive Impairment and Dementia: Role of Tau Protein, beta-Amyloid and LPS in Serum and Curli Protein in Stool. Nutrients. (2023) 12:199. doi: 10.3390/nu15040932, PMID: 36839291 PMC9961602

[63] Popescu C Munteanu C Anghelescu A Ciobanu V Spinu A Andone I . Novelties on neuroinflammation in alzheimer’s disease-focus on gut and oral microbiota involvement. Int J Mol Sci. (2024) 25:11272. doi: 10.3390/ijms252011272, PMID: 39457054 PMC11508522

[64] Ma X Kim JK Shin YJ Son YH Lee DY Park HS . Alleviation of Cognitive Impairment-like Behaviors, Neuroinflammation, Colitis, and Gut Dysbiosis in 5xFAD Transgenic and Aged Mice by Lactobacillus mucosae and Bifidobacterium longum. Nutrients. (2023) 15:3381. doi: 10.3390/nu15153381, PMID: 37571319 PMC10421059

[65] Baek JS Shin YJ Ma X Park HS Hwang YH Kim DH . Bifidobacterium bifidum and Lactobacillus paracasei alleviate sarcopenia and cognitive impairment in aged mice by regulating gut microbiota-mediated AKT, NF-kappaB, and FOXO3a signaling pathways. Immun Ageing. (2023) 20:56. doi: 10.1186/s12979-023-00381-5, PMID: 37872562 PMC10591382

[66] Ma X Yoo JW Shin YJ Park HS Son YH Kim DH . Alleviation of Porphyromonas gingivalis or Its Extracellular Vesicles Provoked Periodontitis and Cognitive Impairment by Lactobacillus pentosus NK357 and Bifidobacterium bifidum NK391. Nutrients. (2023) 15:1068. doi: 10.3390/nu15051068, PMID: 36904068 PMC10005711

[67] Silva YP Bernardi A Frozza RL . The role of short-chain fatty acids from gut microbiota in gut-brain communication. Front Endocrinol (Laus). (2020) 11:25. doi: 10.3389/fendo.2020.00025, PMID: 32082260 PMC7005631

[68] Lee J D’Aigle J Atadja L Quaicoe V Honarpisheh P Ganesh BP . Gut microbiota-derived short-chain fatty acids promote poststroke recovery in aged mice. Circ Res. (2020) 127:453–65. doi: 10.1161/CIRCRESAHA.119.316448, PMID: 32354259 PMC7415518

[69] Fernando W Flint SH Ranaweera K Bamunuarachchi A Johnson SK Brennan CS . The potential synergistic behaviour of inter- and intra-genus probiotic combinations in the pattern and rate of short chain fatty acids formation during fibre fermentation. Int J Food Sci Nutr. (2018) 69:144–54. doi: 10.1080/09637486.2017.1340932, PMID: 28659066

[70] Suzuki T . Regulation of intestinal epithelial permeability by tight junctions. Cell Mol Life Sci. (2013) 70:631–59. doi: 10.1007/s00018-012-1070-x, PMID: 22782113 PMC11113843

[71] Chen Y Zhao N Han S Zhao G Jiang Y Li C . Tricobalt tetraselenide nanoparticles improve intestinal barrier function by reshaping the gut microbiota and fortifying epithelial tight junctions. Biomater Sci. (2025) 13. doi: 10.1039/D5BM00712G, PMID: 40757979

[72] Xiao W Su J Gao X Yang H Weng R Ni W . The microbiota-gut-brain axis participates in chronic cerebral hypoperfusion by disrupting the metabolism of short-chain fatty acids. Microbiome. (2022) 10:62. doi: 10.1186/s40168-022-01255-6, PMID: 35430804 PMC9013454

[73] Chen M Li Y Zhai Z Wang H Lin Y Chang F . Bifidobacterium animalis subsp. lactis A6 ameliorates bone and muscle loss via modulating gut microbiota composition and enhancing butyrate production. Bone Res. (2025) 13:28. doi: 10.1038/s41413-024-00381-1, PMID: 40000617 PMC11862215

[74] Jiang Y Li K Li X Xu L Yang Z . Sodium butyrate ameliorates the impairment of synaptic plasticity by inhibiting the neuroinflammation in 5XFAD mice. Chem Biol Interact. (2021) 341:109452. doi: 10.1016/j.cbi.2021.109452, PMID: 33785315

[75] Wei H Yu C Zhang C Ren Y Guo L Wang T . Butyrate ameliorates chronic alcoholic central nervous damage by suppressing microglia-mediated neuroinflammation and modulating the microbiome-gut-brain axis. BioMed Pharmacother. (2023) 160:114308. doi: 10.1016/j.biopha.2023.114308, PMID: 36709599

[76] Soliman ML Puig KL Combs CK Rosenberger TA . Acetate reduces microglia inflammatory signaling *in vitro*. J Neurochem. (2012) 123:555–67. doi: 10.1111/j.1471-4159.2012.07955.x, PMID: 22924711 PMC3472042

[77] Soliman ML Combs CK Rosenberger TA . Modulation of inflammatory cytokines and mitogen-activated protein kinases by acetate in primary astrocytes. J Neuroimmune Pharmacol. (2013) 8:287–300. doi: 10.1007/s11481-012-9426-4, PMID: 23233245 PMC3587660

[78] Liu J Li H Gong T Chen W Mao S Kong Y . Anti-neuroinflammatory Effect of Short-Chain Fatty Acid Acetate against Alzheimer’s Disease via Upregulating GPR41 and Inhibiting ERK/JNK/NF-kappaB. J Agric Food Chem. (2020) 68:7152–61. doi: 10.1021/acs.jafc.0c02807, PMID: 32583667

[79] Tomova A Bukovsky I Rembert E Yonas W Alwarith J Barnard ND . The effects of vegetarian and vegan diets on gut microbiota. Front Nutr. (2019) 6:47. doi: 10.3389/fnut.2019.00047, PMID: 31058160 PMC6478664

[80] Maslowski KM Vieira AT Ng A Kranich J Sierro F Yu D . Regulation of inflammatory responses by gut microbiota and chemoattractant receptor GPR43. Nature. (2009) 461:1282–6. doi: 10.1038/nature08530, PMID: 19865172 PMC3256734

[81] Tian S Lei Y Zhao F Che J Wu Y Lei P . Improving insulin resistance by sulforaphane via activating the Bacteroides and Lactobacillus SCFAs-GPR-GLP1 signal axis. Food Funct. (2024) 15:8644–60. doi: 10.1039/D4FO01059K, PMID: 39045769

[82] Bai T Xu Z Xia P Feng Y Liu B Liu H . The short-term efficacy of bifidobacterium quadruple viable tablet in patients with diarrhea-predominant irritable bowel syndrome: potentially mediated by metabolism rather than diversity regulation. Am J Gastroenterol. (2023) 118:1256–67. doi: 10.14309/ajg.0000000000002147, PMID: 36717369

[83] Xiao-Hang Q Si-Yue C Hui-Dong T . Multi-strain probiotics ameliorate Alzheimer’s-like cognitive impairment and pathological changes through the AKT/GSK-3beta pathway in senescence-accelerated mouse prone 8 mice. Brain Behav Immun. (2024) 119:14–27. doi: 10.1016/j.bbi.2024.03.031, PMID: 38548184

[84] Zhao S Hu S Sun K Luo L Zeng L . Long-term Pu-erh tea consumption improves blue light-induced depression-like behaviors. Food Funct. (2023) 14:2313–25. doi: 10.1039/D2FO02780A, PMID: 36779860

[85] Livingston DBH Sweet A Rodrigue A Kishore L Loftus J Ghali F . Dietary flaxseed and flaxseed oil differentially modulate aspects of the microbiota gut-brain axis following an acute lipopolysaccharide challenge in male C57Bl/6 mice. Nutrients. (2023) 15:3542. doi: 10.3390/nu15163542, PMID: 37630732 PMC10459276

[86] Zhang K Wang F Zhai M He M Hu Y Feng L . Hyperactive neuronal autophagy depletes BDNF and impairs adult hippocampal neurogenesis in a corticosterone-induced mouse model of depression. Theranostics. (2023) 13:1059–75. doi: 10.7150/thno.81067, PMID: 36793868 PMC9925310

[87] Abdelhamid M Zhou C Jung CG Michikawa M . Probiotic bifidobacterium breve MCC1274 mitigates alzheimer’s disease-related pathologies in wild-type mice. Nutrients. (2022) 14:2543. doi: 10.3390/nu14122543, PMID: 35745273 PMC9231139

[88] Gao L Zhang Y Sterling K Song W . Brain-derived neurotrophic factor in Alzheimer’s disease and its pharmaceutical potential. Transl Neurodegener. (2022) 11:4. doi: 10.1186/s40035-022-00279-0, PMID: 35090576 PMC8796548

[89] Jia M Ning F Wen J Wang X Chen J Hu J . Secoisolariciresinol diglucoside attenuates neuroinflammation and cognitive impairment in female Alzheimer’s disease mice via modulating gut microbiota metabolism and GPER/CREB/BDNF pathway. J Neuroinflamm. (2024) 21:201. doi: 10.1186/s12974-024-03195-4, PMID: 39135052 PMC11320852

[90] Kim CS Cha L Sim M Jung S Chun WY Baik HW . Probiotic supplementation improves cognitive function and mood with changes in gut microbiota in community-dwelling older adults: A randomized, double-blind, placebo-controlled, multicenter trial. J Gerontol A Biol Sci Med Sci. (2021) 76:32–40. doi: 10.1093/gerona/glaa090, PMID: 32300799 PMC7861012

[91] Benarroch EE . Brain-derived neurotrophic factor: Regulation, effects, and potential clinical relevance. Neurology. (2015) 84:1693–704. doi: 10.1212/WNL.0000000000001507, PMID: 25817841

[92] Edelmann E Lessmann V Brigadski T . Pre- and postsynaptic twists in BDNF secretion and action in synaptic plasticity. Neuropharmacology. (2014) 76:610–27. doi: 10.1016/j.neuropharm.2013.05.043, PMID: 23791959

[93] Kim H Shin J Kim S Kim S Cho B Park SJ . Bifidobacterium bifidum BGN4 and Bifidobacterium longum BORI promotes neuronal rejuvenation in aged mice. Biochem Biophys Res Commun. (2022) 603:41–8. doi: 10.1016/j.bbrc.2022.03.024, PMID: 35278878

[94] Xu Z Liu C Wang R Gao X Hao C Liu C . A combination of lycopene and human amniotic epithelial cells can ameliorate cognitive deficits and suppress neuroinflammatory signaling by choroid plexus in Alzheimer’s disease rat. J Nutr Biochem. (2021) 88:108558. doi: 10.1016/j.jnutbio.2020.108558, PMID: 33249184

[95] Hu Y Wang Z Pan S Zhang H Fang M Jiang H . Melatonin protects against blood-brain barrier damage by inhibiting the TLR4/NF-kappaB signaling pathway after LPS treatment in neonatal rats. Oncotarget. (2017) 8:31638–54. doi: 10.18632/oncotarget.15780, PMID: 28404943 PMC5458236

[96] Chen D Li X Zhang L Zhu M Gao L . A high-fat diet impairs mitochondrial biogenesis, mitochondrial dynamics, and the respiratory chain complex in rat myocardial tissues. J Cell Biochem. (2018) 119:9602. doi: 10.1002/jcb.27068, PMID: 30171706 PMC6220867

[97] Balzano T Arenas YM Dadsetan S Forteza J Gil-Perotin S Cubas-Nunez L . Sustained hyperammonemia induces TNF-a IN Purkinje neurons by activating the TNFR1-NF-kappaB pathway. J Neuroinflamm. (2020) 17:70. doi: 10.1186/s12974-020-01746-z, PMID: 32087723 PMC7035786

[98] Hwang CJ Park MH Choi MK Choi JS Oh KW Hwang DY . Acceleration of amyloidogenesis and memory impairment by estrogen deficiency through NF-kappaB dependent beta-secretase activation in presenilin 2 mutant mice. Brain Behav Immun. (2016) 53:113–22. doi: 10.1016/j.bbi.2015.11.013, PMID: 26593275

[99] Kim H Kim S Park SJ Park G Shin H Park MS . Administration of Bifidobacterium bifidum BGN4 and Bifidobacterium longum BORI Improves Cognitive and Memory Function in the Mouse Model of Alzheimer’s Disease. Front Aging Neurosci. (2021) 13:709091. doi: 10.3389/fnagi.2021.709091, PMID: 34421576 PMC8378450

[100] Jang HM Lee KE Kim DH . The Preventive and Curative Effects of Lactobacillus reuteri NK33 and Bifidobacterium adolescentis NK98 on Immobilization Stress-Induced Anxiety/Depression and Colitis in Mice. Nutrients. (2019) 11:819. doi: 10.3390/nu11040819, PMID: 30979031 PMC6521032

[101] Lee HJ Lee KE Kim JK Kim DH . Suppression of gut dysbiosis by Bifidobacterium longum alleviates cognitive decline in 5XFAD transgenic and aged mice. Sci Rep. (2019) 9:11814. doi: 10.1038/s41598-019-48342-7, PMID: 31413350 PMC6694197

[102] Shamsipour S Sharifi G Taghian F . Impact of interval training with probiotic (L. plantarum/Bifidobacterium bifidum) on passive avoidance test, ChAT and BDNF in the hippocampus of rats with Alzheimer’s disease. Neurosci Lett. (2021) 756:135949. doi: 10.1016/j.neulet.2021.135949, PMID: 33974953

[103] Yang Z Zou Y Wang L . Neurotransmitters in prevention and treatment of alzheimer’s disease. Int J Mol Sci. (2023) 24:3841. doi: 10.3390/ijms24043841, PMID: 36835251 PMC9966535

[104] Chen Y Xu J Chen Y . Regulation of neurotransmitters by the gut microbiota and effects on cognition in neurological disorders. Nutrients. (2021) 13:2099. doi: 10.3390/nu13062099, PMID: 34205336 PMC8234057

[105] Zheng X Lu R Pan D Peng L He R Hu Y . Regulatory T and CXCR3+ Circulating tfh cells concordantly shape the neutralizing antibody responses in individuals who have recovered from mild COVID-19. J Infect Dis. (2024) 230:28–37. doi: 10.1093/infdis/jiae061, PMID: 39052730

[106] Holm P Ettrup A Klein AB Santini MA El-Sayed M Elvang AB . Plaque deposition dependent decrease in 5-HT2A serotonin receptor in AbetaPPswe/PS1dE9 amyloid overexpressing mice. J Alzheimers Dis. (2010) 20:1201–13. doi: 10.3233/JAD-2010-100117, PMID: 20413853

[107] Yang Y Zhang L Yu J Ma Z Li M Wang J . A novel 5-HT(1B) receptor agonist of herbal compounds and one of the therapeutic uses for alzheimer’s disease. Front Pharmacol. (2021) 12:735876. doi: 10.3389/fphar.2021.735876, PMID: 34552493 PMC8450432

[108] Busceti CL Di Pietro P Riozzi B Traficante A Biagioni F Nistico R . 5-HT(2C) serotonin receptor blockade prevents tau protein hyperphosphorylation and corrects the defect in hippocampal synaptic plasticity caused by a combination of environmental stressors in mice. Pharmacol Res. (2015) 99:258–68. doi: 10.1016/j.phrs.2015.06.017, PMID: 26145279

[109] Fung TC Vuong HE Luna CDG Pronovost GN Aleksandrova AA Riley NG . Intestinal serotonin and fluoxetine exposure modulate bacterial colonization in the gut. Nat Microbiol. (2019) 4:2064–73. doi: 10.1038/s41564-019-0540-4, PMID: 31477894 PMC6879823

[110] Zhen J Li Y Zhang Y Zhou Y Zhao L Huang G . Shaoyao Decoction reduced T lymphocyte activation by regulating of intestinal flora and 5-hydroxytryptamine metabolism in ulcerative colitis. Chin Med. (2024) 19:87. doi: 10.1186/s13020-024-00958-2, PMID: 38879471 PMC11180410

[111] Zhu S Yu Q Xue Y Li J Huang Y Liu W . Bifidobacterium bifidum CCFM1163 alleviates cathartic colon by activating the BDNF-TrkB-PLC/IP(3) pathway to reconstruct the intestinal nerve and barrier. Food Funct. (2025) 16:2057–72. doi: 10.1039/D4FO05835F, PMID: 39963068

[112] Agnihotri N Mohajeri MH . Involvement of intestinal microbiota in adult neurogenesis and the expression of brain-derived neurotrophic factor. Int J Mol Sci. (2022) 23:15934. doi: 10.3390/ijms232415934, PMID: 36555576 PMC9783874

[113] Tian P O’Riordan KJ Lee YK Wang G Zhao J Zhang H . Towards a psychobiotic therapy for depression: Bifidobacterium breve CCFM1025 reverses chronic stress-induced depressive symptoms and gut microbial abnormalities in mice. Neurobiol Stress. (2020) 12:100216. doi: 10.1016/j.ynstr.2020.100216, PMID: 32258258 PMC7109524

[114] Zhang S Lv S Li Y Wei D Zhou X Niu X . Prebiotics modulate the microbiota-gut-brain axis and ameliorate cognitive impairment in APP/PS1 mice. Eur J Nutr. (2023) 62:2991–3007. doi: 10.1007/s00394-023-03208-7, PMID: 37460822

[115] Petroff OA . GABA and glutamate in the human brain. Neuroscientist. (2002) 8:562–73. doi: 10.1177/1073858402238515, PMID: 12467378

[116] Sallard E Letourneur D Legendre P . Electrophysiology of ionotropic GABA receptors. Cell Mol Life Sci. (2021) 78:5341–70. doi: 10.1007/s00018-021-03846-2, PMID: 34061215 PMC8257536

[117] Treiman DM . GABAergic mechanisms in epilepsy. Epilepsia. (2001) 42:8–12. doi: 10.1046/j.1528-1157.2001.042suppl.3008.x, PMID: 11520315

[118] Li W Liang H He W Gao X Wu Z Hu T . Genomic and functional diversity of cultivated Bifidobacterium from human gut microbiota. Heliyon. (2024) 10:e27270. doi: 10.1016/j.heliyon.2024.e27270, PMID: 38463766 PMC10923715

[119] Koh W Kwak H Cheong E Lee CJ . GABA tone regulation and its cognitive functions in the brain. Nat Rev Neurosci. (2023) 24:523–39. doi: 10.1038/s41583-023-00724-7, PMID: 37495761

[120] Conn KA Borsom EM Cope EK . Implications of microbe-derived ɣ-aminobutyric acid (GABA) in gut and brain barrier integrity and GABAergic signaling in Alzheimer’s disease. Gut Microbes. (2024) 16:2371950. doi: 10.1080/19490976.2024.2371950, PMID: 39008552 PMC11253888

[121] Siragusa S De Angelis M Di Cagno R Rizzello CG Coda R Gobbetti M . Synthesis of gamma-aminobutyric acid by lactic acid bacteria isolated from a variety of Italian cheeses. Appl Environ Microbiol. (2007) 73:7283–90. doi: 10.1128/AEM.01064-07, PMID: 17890341 PMC2168214

[122] Tames H Sabater C Margolles A Ruiz L Ruas-Madiedo P . Production of GABA in milk fermented by Bifidobacterium adolescentis strains selected on the bases of their technological and gastrointestinal performance. Food Res Int. (2023) 171:113009. doi: 10.1016/j.foodres.2023.113009, PMID: 37330847

[123] Casertano M Dekker M Valentino V De Filippis F Fogliano V Ercolini D . Gaba-producing lactobacilli boost cognitive reactivity to negative mood without improving cognitive performance: A human Double-Blind Placebo-Controlled Cross-Over study. Brain Behav Immun. (2024) 122:256–65. doi: 10.1016/j.bbi.2024.08.029, PMID: 39163908

[124] Tomoda T Sumitomo A Shukla R Hirota-Tsuyada Y Miyachi H Oh H . BDNF controls GABA(A)R trafficking and related cognitive processes via autophagic regulation of p62. Neuropsychopharmacology. (2022) 47:553–63. doi: 10.1038/s41386-021-01116-0, PMID: 34341497 PMC8674239

[125] Kolobaric A Andreescu C Jasarevic E Hong CH Roh HW Cheong JY . Gut microbiome predicts cognitive function and depressive symptoms in late life. Mol Psychiatry. (2024) 29:3064–75. doi: 10.1038/s41380-024-02551-3, PMID: 38664490 PMC11449789

[126] Zhou S Ma X Nie H Mu G Wu X . Amelioration of cognitive and behavioral damage by Bifidobacterium breve 05 via regulation of BDNF/NeuN and LPS/Iba1/abeta expression. Int J Food Sci Nutr. (2025) 76:1–16. doi: 10.1080/09637486.2025.2584325, PMID: 41249913

[127] Wu Q Li Q Zhang X Ntim M Wu X Li M . Treatment with Bifidobacteria can suppress Abeta accumulation and neuroinflammation in APP/PS1 mice. PeerJ. (2020) 8:e10262. doi: 10.7717/peerj.10262, PMID: 33194428 PMC7602682

[128] Zhu G Zhao J Wang G Chen W . Bifidobacterium breve HNXY26M4 attenuates cognitive deficits and neuroinflammation by regulating the gut-brain axis in APP/PS1 mice. J Agric Food Chem. (2023) 71:4646–55. doi: 10.1021/acs.jafc.3c00652, PMID: 36888896

[129] Zhu G Zhao J Zhang H Chen W Wang G . Administration of Bifidobacterium breve Improves the Brain Function of Abeta(1-42)-Treated Mice via the Modulation of the Gut Microbiome. Nutrients. (2021) 13:1602. doi: 10.3390/nu13051602, PMID: 34064762 PMC8150793

[130] Wang F Xu T Zhang Y Zheng T He Y He F . Long-term combined administration of Bifidobacterium bifidum TMC3115 and Lactobacillus plantarum 45 alleviates spatial memory impairment and gut dysbiosis in APP/PS1 mice. FEMS Microbiol Lett. (2020) 367:fnaa048. doi: 10.1093/femsle/fnaa048, PMID: 32239209

[131] Abdelhamid M Zhou C Ohno K Kuhara T Taslima F Abdullah M . Probiotic bifidobacterium breve prevents memory impairment through the reduction of both amyloid-beta production and microglia activation in APP knock-in mouse. J Alzheimers Dis. (2022) 85:1555–71. doi: 10.3233/JAD-215025, PMID: 34958017 PMC8925106

[132] Kobayashi Y Sugahara H Shimada K Mitsuyama E Kuhara T Yasuoka A . Therapeutic potential of Bifidobacterium breve strain A1 for preventing cognitive impairment in Alzheimer’s disease. Sci Rep. (2017) 7:13510. doi: 10.1038/s41598-017-13368-2, PMID: 29044140 PMC5647431

[133] Lee DY Shin YJ Kim JK Jang HM Joo MK Kim DH . Alleviation of cognitive impairment by gut microbiota lipopolysaccharide production-suppressing Lactobacillus plantarum and Bifidobacterium longum in mice. Food Funct. (2021) 12:10750–63. doi: 10.1039/D1FO02167B, PMID: 34608923

[134] Yang XQ Zhao Y Xue L Wang HS Zeng J Du JR . Probiotics improve cognitive impairment by decreasing bacteria-related pattern recognition receptor-mediated inflammation in the gut-brain axis of mice. J Integr Neurosci. (2023) 22:92. doi: 10.31083/j.jin2204092, PMID: 37519163

[135] Shamsipour S Sharifi G Taghian F . An 8-Week Administration of Bifidobacterium bifidum and Lactobacillus plantarum Combined with Exercise Training Alleviates Neurotoxicity of Abeta and Spatial Learning via Acetylcholine in Alzheimer Rat Model. J Mol Neurosci. (2021) 71:1495–505. doi: 10.1007/s12031-021-01812-y, PMID: 33715084

[136] Han S Lu Y Xie J Fei Y Zheng G Wang Z . Probiotic gastrointestinal transit and colonization after oral administration: A long journey. Front Cell Infect Microbiol. (2021) 11:609722. doi: 10.3389/fcimb.2021.609722, PMID: 33791234 PMC8006270

[137] Bohnhoff M Drake BL Miller CP . Effect of streptomycin on susceptibility of intestinal tract to experimental Salmonella infection. Proc Soc Exp Biol Med. (1954) 86:132–7. doi: 10.3181/00379727-86-21030, PMID: 13177610

[138] Freter R . The fatal enteric cholera infection in the Guinea pig, achieved by inhibition of normal enteric flora. J Infect Dis. (1955) 97:57–65. doi: 10.1093/infdis/97.1.57, PMID: 13242854

[139] Li L Ma L Fu P . Gut microbiota-derived short-chain fatty acids and kidney diseases. Drug Des Devel Ther. (2017) 11:3531–42. doi: 10.2147/DDDT.S150825, PMID: 29270002 PMC5729884

[140] Martin FP Wang Y Sprenger N Yap IK Lundstedt T Lek P . Probiotic modulation of symbiotic gut microbial-host metabolic interactions in a humanized microbiome mouse model. Mol Syst Biol. (2008) 4:157. doi: 10.1038/msb4100190, PMID: 18197175 PMC2238715

[141] Zhang P Wu X Liang S Shao X Wang Q Chen R . A dynamic mouse peptidome landscape reveals probiotic modulation of the gut-brain axis. Sci Signal. (2020) 13:eabb0443. doi: 10.1126/scisignal.abb0443, PMID: 32723811

[142] Bachem A Makhlouf C Binger KJ de Souza DP Tull D Hochheiser K . Microbiota-derived short-chain fatty acids promote the memory potential of antigen-activated CD8(+) T cells. Immunity. (2019) 51:285–97.e5. doi: 10.1016/j.immuni.2019.06.002, PMID: 31272808

[143] Pei B Peng S Huang C Zhou F . Bifidobacterium modulation of tumor immunotherapy and its mechanism. Cancer Immunol Immunother. (2024) 73:94. doi: 10.1007/s00262-024-03665-x, PMID: 38564002 PMC10987355

[144] Tian P Chen Y Zhu H Wang L Qian X Zou R . Bifidobacterium breve CCFM1025 attenuates major depression disorder via regulating gut microbiome and tryptophan metabolism: A randomized clinical trial. Brain Behav Immun. (2022) 100:233–41. doi: 10.1016/j.bbi.2021.11.023, PMID: 34875345

